# Relationship between periodontal parameters and non-vital pulp in dental clinic patients: a cross-sectional study

**DOI:** 10.1186/s12903-020-01103-9

**Published:** 2020-04-15

**Authors:** Tomotaka Kato, Natsuki Fujiwara, Ryutaro Kuraji, Yukihiro Numabe

**Affiliations:** 1 Department of Oral Health Sciences, School of Dentistry, University of Washington, 8901 Meridian Ave. N, Seattle, WA USA; 2 Division of General Dentistry, Nippon Dental University Hospital, Tokyo, Japan; 3 Fujiwara Dental Clinic, Hiroshima, Japan; 4 Department of Life Science Dentistry, The Nippon Dental University, Tokyo, Japan; 5 Department of Periodontology, The Nippon Dental University School of Life Dentistry at Tokyo, Tokyo, Japan

**Keywords:** Periodontal disease, Non-vital tooth, Obturation, Cross-sectional study, Multiple classification analysis

## Abstract

**Background:**

Endodontic diseases, such as apical periodontitis, communicate with periodontitis and mutually exacerbate them. However, it remains unclear whether pulp condition is a risk factor for periodontal disease. The purpose of this retrospective study was to examine relations between pulp condition and periodontal parameters in Japanese patients who visited a general dental clinic.

**Methods:**

Patients who visited a Japanese general dental clinic from 2016 to 2018 and aged 18 to 81 years were analyzed. Periodontal parameters, tooth condition, and general status of all teeth excluding third molars at the initial visit to the clinic were abstracted. A total of 7105 teeth were analyzed in this study by multiple classification analysis and the Mann–Whitney *U* test. We also performed a sub-analysis of non-vital teeth, which evaluated the presence or absence of unfavorable root canal obturation and apical periodontitis diagnosed by X-ray.

**Results:**

Significant relations between periodontal parameters and non-vital pulp were observed by multiple logistic regression analyses (odds ratio = 1.48; 95% CI = 1.03–2.14) and multiple linear regression analysis (*p* < 0.001). Significant relations between unfavorable root canal obturation tooth with periodontal pocket depth (*p* = 0.00837) and BOP (*p* = 0.0145) were also observed by the Mann–Whitney *U* test.

**Conclusions:**

We demonstrated potential relations between periodontal disease and non-vital pulp.

## Background

Periodontal disease is a common public health concern with prevalences ranging from 40 to 60% [1]. It is one of the etiological factors contributing to tooth loss [2] that is related to an individual’s quality of life [3] and many systemic diseases [4–7]. Thus, periodontal disease control is necessary to maintain both oral and general health. Identification of risk factors for periodontal disease is critical for proper control.

Several risk factors have been identified and categorized as host-specific and environmental factors [8]. The most important risk factor is the microbial biofilm (plaque) [9]. Periodontal disease is initiated and sustained by the dental microbial biofilm. Among host factors, diabetes mellitus is a well-known risk factor of periodontitis [10] [11]. The prevalence of severe periodontitis is increased in patients who have had diabetes mellitus for a long time and poorly-controlled disease [11]. Experience of caries is also reported as a risk factor for periodontitis [12].

As environment factors, smoking is a major risk factor for periodontal disease with estimates ranging between 2.5 and 14.1 (odds ratio) [13]. While many risk factors have been identified, specific tooth risk factors are not as defined except for plaque retention factors, such as ill-fitted prosthetic restorations and irregular tooth morphology.

Recently, a new periodontitis classification system was published by the European Federation of Periodontology and American Academy of Periodontology [14, 15]. This new guideline changed the classification of endo-periodontal lesions. Although the dynamic relationship between pulpal and periodontal tissues was reported, the precise association remains unclear [15]. In addition, some cases of endo-periodontal lesions without periodontal care (only root canal treatment) have been reported [16]. Root canal treatment is recommended as the first choice for endo-periodontal lesions [17]. Moreover, Yoneda et al. also reported the importance of root canal treatment in endo-periodontal lesions [18]. These endo-periodontal lesions are generally refractory and should be classified according to the etiological factors involved, with the most frequent being those occurring in pre-existing periodontal pockets [19].

Therefore, these reports might suggest relations between pulpal and periodontal tissues, and the pulp condition might be a risk factor of periodontal disease. However, to our knowledge, there have been no reports about the relations between the pulp condition and periodontal tissues. The purpose of this retrospective study was to elucidate the relationship between periodontal parameters and the pulp condition such as the presence of non-vital tooth, root canal obturation, and apical periodontitis in Japanese patients who visited a general dental clinic.

## Methods

### Subjects

This investigation was a cross-sectional analysis. Patients aged 18–81 years who first visited a Japanese general dental clinic from 2016 to 2018 were all analyzed retrospectively. Clinical data of all teeth excluding third molars were obtained during oral examinations at initial visits. Exclusion criteria were patients with a fractured tooth, impacted tooth, or tooth stump. Finally, 7105 teeth from 267 patients were analyzed. We excluded patients who did not want to participate in this study via the opt-out method on the dental clinic website and the ethical committee of the Japanese Society of Periodontology approved this clinical cross-sectional study in a matched collective (JSP2019001). To maintain patient anonymity, the personal information relating to the patients of this retrospective study was erased, and participant’s names were replaced by ID numbers.

### Evaluation items

Calibrated dental hygienists performed whole-mouth oral examinations. The manual periodontal probe PCPUNC15 (Hu-Friedy, Chicago, IL, USA) and plaque disclosing solution (Satoh Dental Material, Tokyo, Japan) were used for oral examinations. Oral examinations included periodontal pocket depth (PPD; 6 sites per teeth), bleeding on probing (BOP; 6 sites per teeth), plaque control record (plaque; 4 sites per teeth) [20], and decayed, missing, filled teeth (DFMT) index [12]. One calibrated dentist evaluated the tooth condition for tooth prosthesis and vital or non-vital teeth using X-ray and electric pulp tests with Digitest (Parkell, NY, USA). The general condition and smoking history of participants were also evaluated by medical interview (these data were self-reported by the subjects). Smoking history was categorized by the presence or absence of smoking experience up until the present.

Moreover, we performed a sub-analysis of non-vital. Non-vital teeth were evaluated for the presence or absence of unfavorable root canal obturation (determined as > 2-mm dead space from the root apex) [21, 22] and the presence or absence of apical periodontitis (determined by > 2-mm transmission image) [23, 24], which was diagnosed by an X-ray. General conditions and smoking history were also assessed by interview, and patients who had significant disease that hindered their ability to visit the dental clinic were excluded.

### Statistical analysis

The Mann–Whitney *U* test or chi-squared test was used to assess differences in continuous and categorical variables, respectively, between PPD ≥ 4 mm and PPD < 4 mm groups (year, DMFT, BOP site, and plaque were analyzed by Mann–Whitney U test and sex, smoking, diabetes mellitus, molar, prosthesis, and non-vital pulp were analyzed by chi-squared test). Logistic regression analysis was applied to determine risk factors of deep PPD (≥ 4 mm) in subjects. Odds ratios and 95% confidence intervals (CIs) were used to assess the independent contribution of each identified risk factor (year, sex, smoking, diabetes mellitus, DMFT, molar, prosthesis, plaque and non-vital pulp). Multivariate linear regression analyses were conducted to evaluate the relation between patient characteristics (year, sex, smoking, diabetes mellitus, DMFT, molar, prosthesis, plaque and non-vital pulp) and PPD or BOP. Lastly, sub-analysis was carried out in non-vital teeth. Relations between the sub-class of non-vital tooth (root canal obturation and apical periodontitis) and periodontal parameter (PPD and BOP) were assessed using the Mann–Whitney *U* test. Statistical analyses were performed with EZR (Saitama Medical Center, Jichi Medical University, Saitama, Japan) [25], which is a graphical user interface for the open source statistical program “R” (The R Foundation for Statistical Computing, Vienna, Austria). More precisely, it is a modified version of R commander designed to add statistical functions frequently used in biostatistics. Statistical significance was set at *p* < 0.05.

## Results

### Study population

A total of 267 participants and 7105 teeth were analyzed by multiple classification analysis. Table 1 shows the characteristics of patients classified by PPD (≥ 4 mm and < 4 mm). Mean ages of PPD ≥ 4 mm and PPD < 4 mm groups were 44.35 and 39.09 years, respectively. Sex, smoking history, diabetes mellitus, tooth type, prosthesis, moving score, BOP, plaque, and non-vital pulp were significantly different between the two groups (*p* < 0.001). However, DMFT was not significantly different between the two groups. The percentage of non-vital teeth in the PPD ≥ 4 mm group (12.2%) was twice that in the PPD < 4 mm group (6.2%).

**Table 1:** The characteristics of tooth in the PD≧4 mm and PD < 4 mm groups

	PD < 4 ***n*** = 6092	PD ≧ 4 ***n*** = 1013	***p-***value
Year mean (sd)	39.09 (13.85)	44.35 (13.34)	< 0.001
Sex women n(%)	3740 (61.4)	467 (46.1)	< 0.001
Smoking n(%)	1285 (21.1)	485 (47.9)	< 0.001
Diabetes mellitus n(%)	55 (0.9)	31 (3.1)	< 0.001
DMFT mean (sd)	10.63 (6.46)	10.85 (6.62)	0.335
Molar n (%)	1437 (23.6)	552 (54.5)	< 0.001
Prosthesis n (%)	361 (5.9)	125 (12.3)	< 0.001
BOP site mean (sd)	0.73 (1.19)	2.30 (1.80)	< 0.001
Plaque mean (sd)	0.97 (1.26)	1.31 (1.51)	< 0.001
Pulp non-vital n(%)	377 (6.2)	124 (12.2)	< 0.001

Year mean = mean of the patient’s year which had subject’s tooth, Sex women = number of teeth which were refer women. Smoking = number of teeth which were refer smoking experience of the patients, Diabetes mellitus = number of teeth which refer diabetes mellitus patients, DMFT mean = mean of the DMFT index which patient had subject’s tooth, Molar = number of tooth which was molar, Prosthesis = number of tooth which was restored by crown or bridge as prosthesis, BOP site = mean of BOP site of the subject’s tooth, Plaque = mean of plaque site of the subject’s tooth, Pulp non- vital = number of tooth which was non-vital teeth

### Variables associated with periodontal status by multivariate analysis

Multiple logistic regression analyses (Table 2) showed that PPD ≥ 4 mm was associated with year (odds ratio = 1.03; 95% CI = 1.02–1.03), female sex (odds ratio = 0.79; 95% CI = 0.63–0.92), smoking history (odds ratio = 3.21; 95% CI = 2.73–3.76), diabetes mellitus (odds ratio = 1.97; 95% CI = 1.20–3.23), DMFT index score (odds ratio = 0.97; 95% CI = 0.96–0.99), molar (odds ratio = 4.28; 95% CI = 3.69–4.97), plaque (odds ratio = 1.13; 95% CI = 1.07–1.19), and non-vital pulp (odds ratio = 1.48; 95% CI = 1.03–2.14).

**Table 2:** Risk factors of deep periodontal pocket by logistic regression analysis

Variable	Odds ratio	95% CI	***p-***value
Intercept	0.025	0.019, 0.033	< 0.001
Year	1.030	1.020, 1.030	< 0.001
Sex (women)	0.787	0.673, 0.919	0.002
Smoking	3.210	2.730, 3.760	< 0.001
Diabetes mellitus	1.970	1.200, 3.230	0.007
DMFT	0.972	0.960, 0.985	< 0.001
Molar	4.280	3.690, 4.970	< 0.001
Prosthesis	1.010	0.693, 1.470	0.969
Plaque	1.130	1.070, 1.190	< 0.001
Pulp non-vital	1.480	1.030, 2.140	0.035

Among the maximal PPD, there were significant associations with year (p < 0.001), female sex (p < 0.001), smoking history (p < 0.001), DMFT index score (*p* = 0.005), molar (p < 0.001), plaque (p < 0.001), and non-vital pulp (p < 0.001) (Table 3).

**Table 3:** Multiple linear regression of periodontal pocket by different parameters

Variable	Estimate	95% CI	Standard error	***p-***value
Intercept	2.0152	1.930, 2.101	0.044	< 0.001
Year	0.0072	0.005, 0.009	0.001	< 0.001
Sex (women)	−0.2143	− 0.266, − 0.163	0.026	< 0.001
Smoking	0.4402	0.381, 0.499	0.030	< 0.001
Diabetes mellitus	0.0909	−0.131, 0.313	0.113	0.421
DMFT	−0.0062	−0.011, − 0.002	0.002	0.005
Molar	0.8866	0.832, 0.941	0.028	< 0.001
Prosthesis	0.1275	−0.022, 0.277	0.077	0.0954
Plaque	0.0641	0.046, 0.083	0.009	< 0.001
Pulp non-vital	0.2462	0.101, 0.392	0.074	< 0.001

In addition, multiple linear regression analysis indicated similar significant associations between BOP site and periodontal parameters as observed for maximal PPD (Table 4). In particular, non-vital pulp was also associated with BOP site (*p* = 0.002).

**Table 4:** Multiple linear regression of BOP sites by different parameters

Variable	Estimate	95% CI	Standard error	***p-***value
Intercept	1.0331	0.923, 1.144	0.056	< 0.001
Year	−0.0084	−0.011, − 0.006	0.001	< 0.001
Sex (women)	−0.2497	−0.316, − 0.183	0.034	< 0.001
Smoking	0.4042	0.328, 0.481	0.039	< 0.001
Diabetes mellitus	−0.2363	−0.523, 0.050	0.146	0.106
DMFT	−0.00001	−0.006, 0.006	0.003	0.997
Molar	0.6199	0.549, 0.691	0.036	< 0.001
Prosthesis	0.0309	−0.163, 0.225	0.099	0.755
Plaque	0.1053	0.081, 0.129	0.012	< 0.001
Pulp non-vital	0.2948	0.107, 0.483	0.096	0.002

### Sub-analysis of non-vital teeth

In the sub-analysis of non-vital teeth, significant associations were found between root canal obturation (favorable obturation vs unfavorable obturation) and PPD (*p* = 0.00837) and BOP site (*p* = 0.0145) by the Mann–Whitney U test (Table 5). However, no significant relationships were observed with the presence or absence of apical periodontitis.

**Table 5:** Sub-analysis in non-vital tooth, the relation of periodontal pocket and BOP sites categorized by the condition of root canal obturation and apical periodontitis

Variable	Periodontal pocket depth (SD)	***p-***value	BOP sites (SD)	***p-***value
Root canal obturation		0.00837		0.0145
Favorable obturation	2.99 (1.34)		1.36 (1.62)	
Unfavorable obturation	3.25 (1.38)		1.69 (1.69)	
Apical periodontitis		0.129		0.561
Absence	3.04 (1.32)		1.45 (1.62)	
Presence	3.24 (1.46)		1.57 (1.74)	

## Discussion

In this retrospective study, we analyzed relations between periodontal parameters and pulp conditions using data collected during oral examinations at a general dental clinic. We confirmed that age, smoking history, sex, diabetes mellitus, and plaque were significantly associated with periodontal parameters, as indicated by many previous studies. According to logistic regression analysis and multiple linear regression analysis, significant relations between periodontal parameters and non-vital pulp were also observed.

Furthermore, we performed the sub-analysis of non-vital teeth to clarify relations between periodontal parameters and pulp conditions. Interestingly, there were significant relationships between PPD and BOP with root canal obturation, but not apical periodontitis. These results suggested that poor root canal obturation might be a new risk factor of periodontal disease. Similar findings were reported for dental implants [26]. Peri-implantitis is included as periodontal disease and has recently gained attention because implant therapy is not yet standardized [27]. Risk factors of peri-implantitis are quite similar to those of periodontal disease [28]. In addition, it was reported that a micro-gap between the abutment and implant is a risk factor unique to dental implants that is associated with reduced peri-implant inflammatory cell accumulation and minimal bone loss [26, 29]. Therefore, this space at the abutment–implant interface might be a source of microbial infection.

In periodontal disease, an area similar to the microgap may exist between the non-vital pulp cavity and periodontal tissue. Structures such as the lateral branch, microcrack, and dental tubules have been considered. In particular, some studies indicated that dental tubules could serve as an entry point for bacteria between periodontal tissue and dental pulp [30, 31]. In this study, we found relations between periodontal parameters and non-vital teeth, especially poor root canal obturation, which seem to support these previous results.

However, Xavier et al. reported that there was a relation between periodontal disease and apical periodontitis development [32]. The authors also reported communication between periodontal tissue and dental pulp through dental tubules (odd ratio = 5.19 compared with presence or absence of periodontal disease). In our sub-analysis of non-vital teeth, there was no association between the presence or absence of apical periodontitis and PPD or BOP site, however, the group that had apical periodontitis exhibited worse periodontal parameters than the group that did not have apical periodontitis (Table 5). One potential explanation for the discrepancy in results between their study and ours is the inclusion criteria. Namely, we evaluated all teeth (excluding third molars) of all patients included in analyses, whereas only 1 tooth per patient was included in Xavier et al.’s study.

We analyzed relations between many characteristics and periodontal parameters. Results of multiple classification analysis showed significant relations of age, sex, smoking history, diabetes mellitus, plaque, and molar with periodontal parameters. These results were similar to those of previous studies [8, 33], validating our mode of analysis.

There are some limitations to the present study. First, because we conducted a retrospective cross-sectional study, we could not explain characteristics as risk factors of periodontal disease or evaluate risk ratios. Second, determination of vital or non-vital pulp was sometimes difficult because of self-reporting except for cases diagnosed by X-ray. However, we used electric pulp tests [34] in cases that were difficult to determine and attempted to be as objective as possible. Thus, future prospective studies are needed to evaluate the risk ratio of non-vital pulp related to periodontal disease.

## Conclusion

The present study demonstrated significant relations between teeth with non-vital pulp and poor periodontal parameters. Moreover, significant associations between periodontal parameters and root canal obturation, but not apical periodontitis, were observed. Therefore, our findings suggest that there is a relationship between the condition of the dental pulp and periodontal disease.

## Data Availability

The datasets used and analyzed in this study are available on reasonable request from the corresponding author.

## Abbreviations

QOL: quality of life
BOP: bleeding on probing
PPD: periodontal pocket depth
